# Pneumothorax/pneumomediastinum as a complication of foreign body inhalation in 3 pediatric patients: A case series

**DOI:** 10.1097/MD.0000000000031073

**Published:** 2022-10-14

**Authors:** Ahmed K. Alahmari, Abdullah A. Alhelali, Abdullah K. Alahmari, Nehad J. Ahmed, Assaf A. Alkathiri, Khalid T. Ardi, Mohammed H. Baali, Musleh H. Mubarki, Mohammed A. Alhamoud

**Affiliations:** a Otolaryngology Department, Aseer Central Hospital, and Abha Children Hospital, Abha, Saudi Arabia; b Clinical Pharmacy Department, College of Pharmacy, Prince Sattam Bin Abdulaziz University, AL-Kharj, Saudi Arabia.

**Keywords:** case series, foreign body aspiration, pneumomediastinum, Saudi Arabia

## Abstract

Foreign body inhalation (FBI) is a serious and common emergency in children. Such children present in the emergency room (ER) with cough, shortness of breath, choking, or wheezing but rarely present with pneumomediastinum.

**Patient concerns:**

Three children aged 2 to 5 years (2 girls and 1 boy) were seen in our ER complaining of FBI. Emergency bronchoscopy removal of the inhaled foreign body was performed; however, all 3 patients developed pneumomediastinum.

**Diagnosis:**

A foreign body inhalation complicated by Pneumomediastinum/pneumothorax.

**Intervention and outcomes:**

All the patients underwent emergency bronchoscopy and foreign body removal. After the ER intervention, 2 children were placed in the pediatric intensive care unit, and the pneumomediastinum resolved without intervention. The third patient required an operation for chest tube placement, which was then observed in the pediatric intensive care unit, and had several chest radiography follow-ups. After 5 days, the patient exhibited clinical improvement, and the chest tube was removed.

**Conclusion:**

In this case series, we present 3 cases of children aged 2 to 5 years seen in our ER with a history of different types of organic FBI complicated by pneumomediastinum/pneumothorax. Pneumomediastinum/pneumothorax is a rare complication of FBI in pediatric patients. However, such complications require multidisciplinary collaboration for early diagnosis and intervention.

## 1. Introduction

Pneumomediastinum or mediastinal emphysema is a serious respiratory condition of abnormal air leakage into the mediastinal cavity.^[1–4]^ If not fully treated, pneumomediastinum can result in devastating consequences, including pneumothorax,^[2]^ pneumopericardium, pneumoperitoneum, pneumoretroperitoneum,^[4]^ and subcutaneous emphysema.^[5]^ Previous literature has identified that patients with asthma, diabetes, chest trauma, or lung cancer; those who exercise vigorously; those who gave birth; smokers; drug abusers; and those who experienced excessive coughing or vomiting had a higher risk of pneumomediastinum development.^[1,2,6,7]^ Pneumomediastinum can be categorized into 2 main types: spontaneous pneumomediastinum and secondary pneumomediastinum.^[1,5]^ Spontaneous pneumomediastinum indicates the presence of pneumomediastinum in generally healthy individuals with no history of chest trauma, lung operations, injuries, or diseases.^[1–3,5,6]^ On the other hand, secondary pneumomediastinum is considered if any of the underlying causes (iatrogenic, traumatic, non-traumatic) for air leaking into the mediastinum is identified.^[1]^ Pneumomediastinum is a rare condition with varying incidences according to individual age, sex, and other factors. Some previous studies have reported an incidence of 1.7 to 2.5 per 1000 infants^[5]^ and between 1 per 8000 and 1 per 15,000 among pediatric patients.^[1,5]^ Furthermore, men showed a higher tendency to develop pneumomediastinum in all age groups than women.^[5]^ Generally, pneumomediastinum is uncommon among children, and the most common causes among children are asthma exacerbation^[8,9]^ and, very rarely, foreign body aspiration (FBA).^[9]^

According to Yang et al, children are more vulnerable to frequent and sometimes fatal conditions known as FBA^[10]^ This condition is considered 1 of the leading causes of hospital admission and immediate lifesaving operations performed by otorhinolaryngologists.^[11]^ In the USA, it was estimated that FBA is associated with 500 mortalities among children each year and is linked to more than 40% of the total fatal incidents in children younger than 1 year of age.^[12]^ Patient presentations are usually different; however, most patients present with coughing, choking, wheezing, shortness of breath, recurrent pneumonia, obstructive emphysema, and respiratory distress.^[10,11]^ In practice, removal of a foreign body through tracheobronchoscopy can lead to secondary pneumomediastinum and/or subcutaneous emphysema. However, pneumomediastinum as a complication of the foreign body itself has been infrequently reported.^[11]^ Therefore, this study aimed to present a rare complication of pneumomediastinum due to FBA.

## 2. Case reports

The study protocol of the current case series was reviewed and approved by the Asser Regional Committee for Research Ethics, Ministry of Health, Kingdom of Saudi Arabia (REC-No 06-07-2021 and approval from April 08, 2021). All parents of the children included in this study provided written informed consent for participation and publication.

### 2.1. Case 1

**Figure 1.**
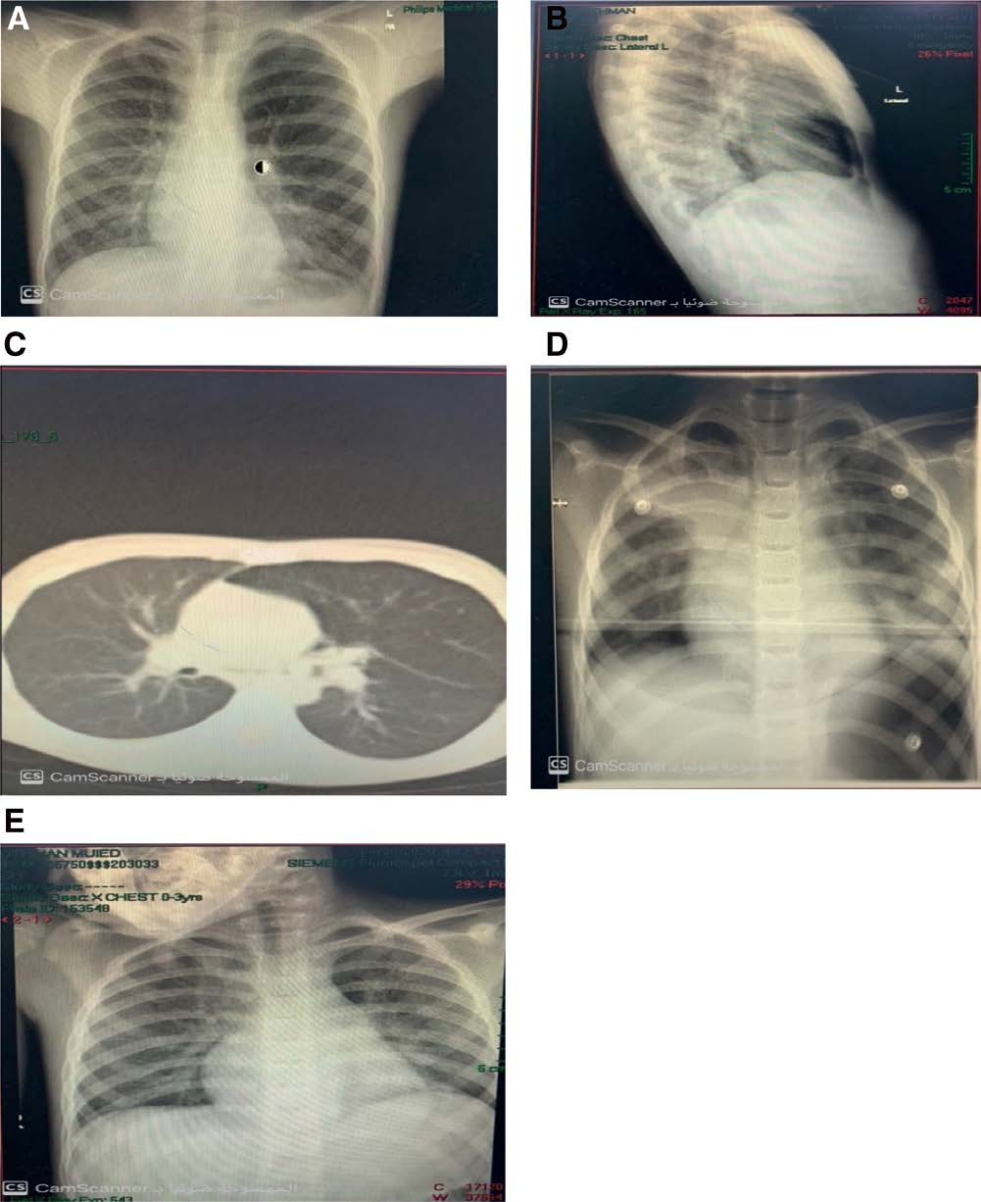
Chest radiography and CT scan were performed for case 1 before, during, and after FB removal. (A) Preoperative chest radiograph AP view. (B) Preoperative lateral chest radiograph view. (C) Axial CT chest with pneumomediastinum. (D) Intraoperative chest radiograph. (E) Postoperative 6 hours chest radiograph. CT = computed tomography.

The first patient was a 5-year-old boy who visited our emergency room (ER) with an unclear history of foreign body inhalation 4 months before the ER visit. His family reported that he complained of recurrent chest infection, chronic coughing, dyspnea, and recent chest pain. His medical records revealed no chronic illnesses. The patient showed no improvement with bronchodilators, and antibiotics were administered in the ER; however, coughing and central chest aching persisted. Upon examination, the patient looked well, with an oxygen saturation level of 92% on room air, decreased air entry into the right lung with bilateral wheezing, and no signs of surgical emphysema. Foreign body inhalation was suspected on chest radiography; thus, computed tomography (CT) was performed and revealed left-sided lung hyperinflation with pneumomediastinum. The patient was then admitted to the hospital where general work-up, steroids, and antibiotics were initiated, and the patient was prepared for bronchoscopy. Bronchoscopy was performed under general anesthesia and a foreign body (peanut) was detected in the left main bronchus (Fig. 1A–E). Urgent intraoperative chest radiography showed no pneumomediastinum progression. Following the operation, the patient was extubated and admitted to the pediatric intensive care unit (PICU) for 1 day under observation and chest radiography every 6 hours. As the patient improved, he was transferred to the general ward for 2 days and was discharged.

### 2.2. Case 2

**Figure 2.**
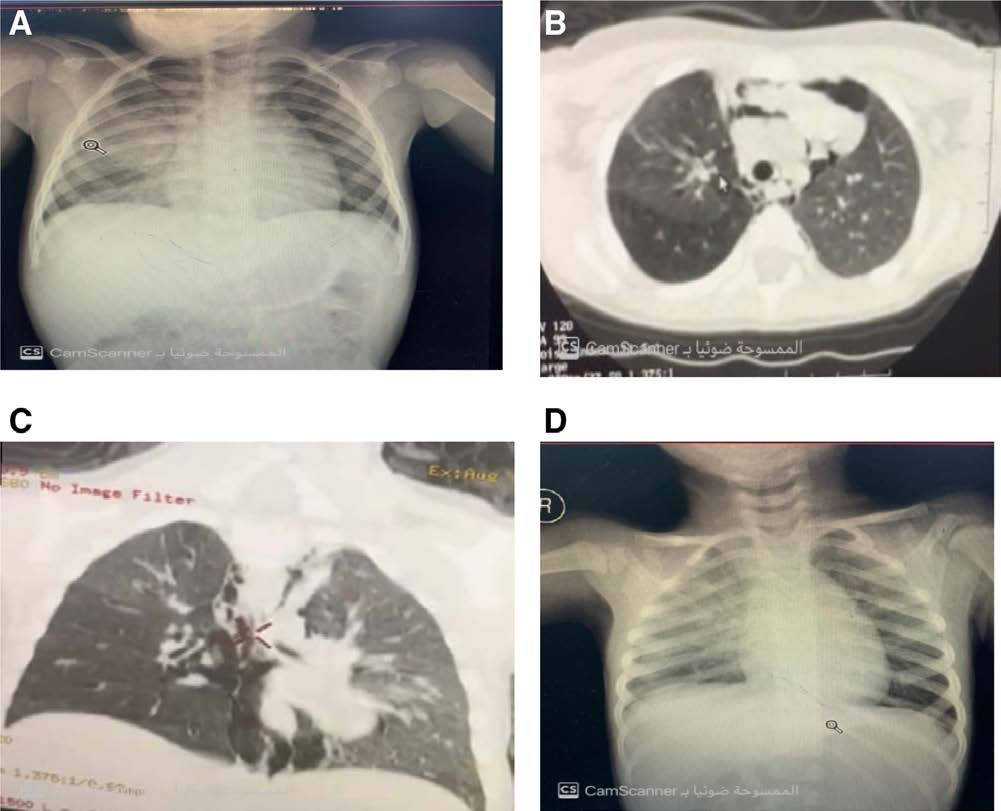
Chest radiography and CT scan were performed for case 2 before, during, and after FB removal. (A) AP chest radiograph upon patient’s arrival to the ER. (B) Axial view of the chest CT with pneumomediastinum. (C) Coronal view of the chest CT with pneumomediastinum. (D) Postoperative chest radiograph AP view. CT = computed tomography.

The second case involved a 2-year-old girl with a 1-day history of foreign body inhalation associated with cyanosis, choking, and apnea caused by continuous coughing. The patient was seen in a peripheral hospital, where she underwent a chest CT scan that showed pneumomediastinum due to suspected foreign body inhalation. Chest radiographs and CT scans before bronchoscopy are shown in Figure 2A–D.

The patient was transferred to our hospital for a lifesaving intervention. Upon evaluation, the patient’s condition was generally good, with an oxygen saturation level of 86% in room air and 93% with a face mask at 2 L/min. Mild surgical emphysema and wheezing were observed on the right side of the chest. On the basis of these observations, the patient underwent urgent preparation for the operating room.

Bronchoscopy revealed a foreign body (seed) in the main right bronchus. An intraoperative chest radiograph showed no expansion of the pneumothorax; thus, the patient was extubated and observed in the PICU for 2 days. Follow-up chest radiography revealed that the patient had recovered well, and she was transferred to the general ward for 1 day. Subsequently, the patient was discharged in good condition.

### 2.3. Case 3

**Figure 3.**
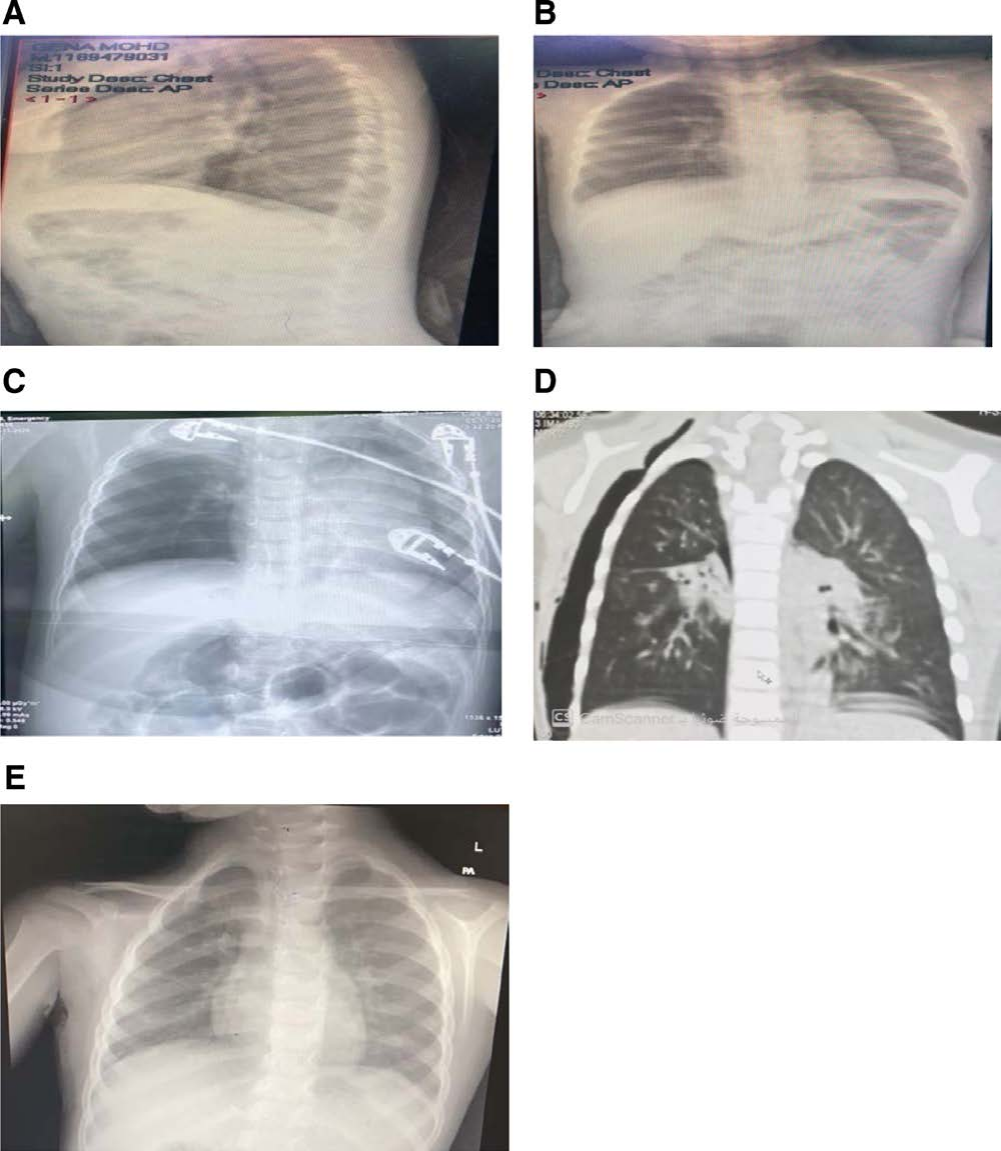
Chest radiography and CT scan were performed for case 3 before, during, and after FB removal. (A) Preoperative lateral chest radiograph. (B) Preoperative AP chest radiograph. (C) Intraoperative chest radiograph showing right lung collapse and pneumothorax. (D) Coronal view of the chest CT showing subcutaneous emphysema. (E) Postoperative and post-ICD removal chest radiograph. AP = anteroposterior, CT = computed tomography, ER = emergency room, FB = foreign body, ICD = Intercostal drainage tube.

The third patient was an 18-month-old girl brought by her family to a peripheral hospital with a history of sudden coughing, choking, and cyanosis that had lasted for a few seconds. On arrival at a peripheral hospital, the patient was normal and had no signs of respiratory distress. Her vital signs were stable, and she had a normal chest radiograph. The patient was referred to our hospital the same day. Upon arrival, the patient appeared normal with no stridor or respiratory distress. Chest evaluation revealed equal bilateral air entry, with no adventitious sounds. After a discussion with her family, she was scheduled for outpatient follow-up for observation and a possible diagnostic bronchoscope. However, she returned to the ER complaining of decreased intake, chest pain, and a severe cough. Upon examination, the patient’s vital signs were stable, with an oxygen saturation level of 92% on room air. She had no stridor, but signs of surgical emphysema in the neck were observed with decreased air entry into the right lung and wheezing on the right side. Chest radiography was performed, and a lateral view of the chest radiograph revealed pneumomediastinum. The patient was immediately prepared for bronchoscopy and a pediatric surgeon was consulted regarding the need for chest tube insertion (Fig. 3A–E).

During bronchoscopy (intraoperative), the patient tended to desaturate quickly, even before the trial of the first-look bronchoscope. Therefore, intubation and ventilation were required. Intraoperative chest radiography was performed before chest tube insertion, and we noticed a collapse of the right lung. Bronchoscopy revealed a foreign body (peanut) on the right lung. Thus, an intercostal drainage tube (ICD) was inserted on the right side and bronchoscopy was performed to remove the foreign body from the right main bronchus. Postoperatively, the patient was transferred to the PICU for 7 days, where she was kept intubated for 5 days and on an ICD for 7 days.

On the second day in the PICU, a chest CT scan was performed, which showed pneumothorax of the right lung. After removal of the ICD on day 7, another chest radiograph was performed to confirm improvement, and patient was discharged in good condition.

## 3. Discussion

In the present case series, we present 3 cases of children aged 2 to 5 years. These children presented to our ER with a history of aspiration of different types of organic foreign bodies complicated by pneumomediastinum. This condition is rare and requires early diagnosis and intervention. Kouritas et al reported that pneumomediastinum is a rare condition that occurs when air leaks into the mediastinum.^[1]^ Meireles et al stated that pneumomediastinum affects between 1 in 7000 and 1 in 45,000 people who are admitted to the hospital.^[13]^ Pneumomediastinum is commonly caused by medical conditions such as asthma, chronic lung disease, infections, and mechanical ventilation,^[14]^ but it is rarely caused by FBA, as shown in previous studies.^[3,4,8–11,15–19]^

In the present case series, 2 patients were girls aged 2 and 1 year, and 1 was a boy aged 5 years. Kouritas et al reported that pneumomediastinum is more likely to occur in children or babies because their chest consists of more cartilage and softer tissue than adults, and it is more common in boys than in girls.^[1]^ Lee et al reported that approximately 77.8% of the patients who had spontaneous pneumomediastinum were boys and 22.2% were girls and that the age of these patients ranged from 9 months to 18 years.^[3]^ Hu et al stated that the most common causes of spontaneous pneumomediastinum are asthma and infections and that the possibility of FBA should be considered, particularly in patients under 3 years of age, because of multiple physiological, environmental, and developmental factors.^[20]^ Furthermore, Semerkant et al reported that non-traumatic pneumomediastinum is mainly caused by asthma; nonetheless, FBA should be considered in children younger than 3 years of age.^[19]^ Altuntaş et al reported that FBA should be investigated in all children, especially those under the age of 3 years and those presenting with pneumomediastinum.^[21]^ Burton et al reported that in children less than 2 years of age with no history of trauma, radiographic findings of pneumomediastinum should prompt further investigation for FBA.^[17]^

Because children have a natural tendency to put items in their mouths, FBA is rather prevalent among kids. FBA has never seemed to decrease in occurrence, and it continues to be a significant cause of death and morbidity in children less than 3 years old.^[22]^ According to the Centers for Disease Control and Prevention, organic food ingredients are the most frequently inhaled or aspirated foreign substances.^[23]^ Pan et al studied 316 kids under the age of 3 who had a documented case of FBA. In their study, they found that nuts were the most aspirated food item, followed by seeds (54.1% vs. 19.6%, respectively).^[24]^ Of the 22 children studied by Sai Akhil et al, 63.6% of the foreign bodies removed during bronchoscopies were found to be composed of organic components, with peanuts being the most often (31.8%).^[25]^ Fidkowski et al found that most aspirated foreign bodies among children were formed of organic materials in a review of the literature on tracheobronchial foreign bodies. Research consistently shows that peanuts and other nuts, as well as seeds like sunflower and watermelon, were the most commonly aspirated foreign bodies.^[26]^

Pneumomediastinum as a complication of FBA is a very rare condition among pediatrics. In fact, the incidence of pediatric secondary pneumomediastinum due to FBA ranges from 1.5% to 2.5%.^[27]^ Burton et al performed a 35-year longitudinal retrospective study of 155 children who presented with FBA and discovered that 10 of them (6.45%) had acquired secondary pneumomediastinum. Peanuts were observed in more than 46% of all cases. Interestingly, all those 10 pneumomediastinum cases were secondary to an organic FBA, with peanuts being the most prevalent type (7 cases).^[17]^ Thirty-nine (1.5%) cases of pneumomediastinum as a complication of FBA was also found in a study by Yang et al, who retroactively reviewed a larger sample of 2643 Chinese children who had FBA between 2010 and 2015. Similar to the previous study, peanuts were the most commonly observed foreign body (59%), followed by other nuts (20.5%).^[10]^ Several published case reports have linked peanuts aspiration to some pediatric pneumomediastinum cases.^[28–30]^ Similarly, 2 of the 3 cases presented in our study were linked to peanuts aspiration. Such finding suggests that peanuts, in particular, are the most common cause of secondary pneumomediastinum in children, suggesting that these nuts should either be away from children’s reach or consumed under parental supervision.

In these 3 cases, Foreign body inhalation was associated with several symptoms. The first case of a 5-year-old patient presented with coughing, dyspnea, and chest pain. The 2-year-old girl in The second case presented with cyanosis, choking, and apnea associated with continued coughing, and the 18-month-old girl in the third case had a history of sudden coughing, choking, and cyanosis. Generally, patients who have experienced pneumomediastinum report symptoms such as chest pain, throat pain, difficulty breathing, a change in voice, or difficulty swallowing.^[31]^ 1 study in Taiwan showed that approximately 81% of spontaneous pneumomediastinum in children was preceded by cough.^[32]^ Wani et al reported that tracheobronchial FBA is a life-threatening emergent condition in children characterized by wheezing, cough, and dyspnea at varying degrees.^[18]^ Furthermore, Yang et al stated that pneumomediastinum secondary to FBA could be life-threatening in some patients. They also said that patients who experience pneumomediastinum secondary to FBA have different degrees of dyspnea and should be evaluated and treated appropriately.^[10]^ Marchiori et al reported that pneumomediastinum is characterized by the presence of air or gas in the mediastinum and can cause dyspnea, chest pain, crackles, and soft tissue emphysema.^[2]^ Gasser et al stated that spontaneous pneumomediastinum is uncommon in children but must be considered in pediatric patients with acute chest and/or neck pain.^[5]^

In the present case series, 3 children presented at the ER with a history of organic foreign body inhalation complicated by pneumomediastinum. All the patients underwent emergency bronchoscopy and foreign body removal. After implementing the ER intervention, 2 children were placed in the PICU, and their pneumomediastinum resolved without intervention. However, the third patient required surgery for chest tube placement, was observed in the PICU, and had several chest radiographic follow-ups. After 5 days, the patient showed clinical improvement, and the chest tube was removed.

Pneumomediastinum is a benign self-limiting condition.^[33]^ There are no reports of fatal outcomes in patients with spontaneous pneumomediastinum in the absence of underlying diseases in the more recent literature, but the prognosis could be worse in the presence of other comorbid conditions.^[33]^ Lee et al reported that approximately 61.1% of patients who had spontaneous pneumomediastinum required intensive care because of respiratory distress.^[3]^ Gasser et al reported that in pediatric spontaneous pneumomediastinum, most patients are hospitalized (88.3%), and their treatment is based on oxygen therapy, painkillers, and rest. They also reported that approximately 25.8% of patients require intensive care and 5.5% require drainage of associated pneumothorax.^[5]^ Moreover, Gasser et al stated that in the absence of other medical conditions, patients’ outcomes are good and there is no need for further chest radiography follow-ups.^[5]^ Furthermore, Koullias et al reported that pneumomediastinum treatment is directed toward symptom relief after the diagnostic approach has excluded significant pathology.^[34]^ Banki et al reported that young patients with spontaneous pneumomediastinum can be managed with a short period of observation and symptomatic treatment, generally without the need for admission or further diagnostic studies.^[6]^ Meireles et al reported that although symptoms such as chest pain and shortness of breath can be frightening, pneumomediastinum usually is not serious, and spontaneous pneumomediastinum often improves by itself.^[13]^ Yang et al reported that pneumomediastinum secondary to FBA is generally benign but may be fatal in some cases.^[10]^

## 4. Conclusion

Pneumomediastinum and thoracic complications rarely present with foreign body inhalation in pediatric patients. However, multidisciplinary teams (otolaryngologists, anesthesiologists, pediatric surgeons, and portable radiography technicians) are required for correct diagnosis and immediate intervention. We emphasize the importance of establishing a protocol or algorithm to address such conditions.

## Author contributions

**Conceptualization:** Ahmed K. Alahmari, Assaf A. Alkathiri, Khalid T. Ardi, Mohammed H. Baali, Musleh H. Mubarki, and Mohammed A. Alhamoud.

**Data curation:** Ahmed K. Alahmari, Khalid T. Ardi, and Mohammed A. Alhamoud.

**Investigation:** Abdullah A. Alhelali, Ahmed K. Alahmari, Assaf A. Alkathiri, and Musleh H. Mubarki.

**Literature search and review:** Abdullah K. Alahmari, Ahmed K. Alahmari, and Nehad J. Ahmed.

**Project administration:** Abdullah A. Alhelali, Nehad J. Ahmed, and Abdullah K. Alahmari.

**Resources:** Ahmed K. Alahmari.

**Supervision:** Abdullah A. Alhelali and Abdullah K. Alahmari.

**Writing – original draft:** Ahmed K. Alahmari, Assaf A. Alkathiri, Khalid T. Ardi, and Mohammed H. Baali.

**Writing – review & editing:** Abdullah K. Alahmari, and Nehad J. Ahmed.
